# Stability of DL-Glyceraldehyde under Simulated Hydrothermal Conditions: Synthesis of Sugar-like Compounds in an Iron(III)-Oxide-Hydroxide-Rich Environment under Acidic Conditions

**DOI:** 10.3390/life12111818

**Published:** 2022-11-08

**Authors:** Claudio Alejandro Fuentes-Carreón, Jorge Armando Cruz-Castañeda, Eva Mateo-Martí, Alicia Negrón-Mendoza

**Affiliations:** 1 Posgrado en Ciencias de la Tierra, Instituto de Ciencias Nucleares, Universidad Nacional Autónoma de México, Mexico City 04510, Mexico; 2 Instituto de Ciencias Nucleares, Universidad Nacional Autónoma de México, Mexico City 04510, Mexico; 3 Centro de Astrobiología (CAB) CSIC-INTA, Ctra. de Ajalvir km 4, 28850 Torrejón de Ardoz, Spain

**Keywords:** chemical evolution, sugar-like compound synthesis, hydrothermal systems

## Abstract

Researchers have suggested that the condensation of low-molecular-weight aldehydes under basic conditions (e.g., pH > 11) is the prebiotic reaction responsible for the abiotic formation of carbohydrates. It has also been suggested that surface hydrothermal systems were ubiquitous during the early Archean period. Therefore, the catalysis of prebiotic carbohydrate synthesis by metallic oxide minerals under acidic conditions in these environments seems considerably more probable than the more widely hypothesized reaction routes. This study investigates the stability of DL-glyceraldehyde and its reaction products under the simulated conditions of an Archean surface hydrothermal system. The Hveradalur geothermal area in Iceland was selected as an analog of such a system. HPLC-ESIMS, UV–Vis spectroscopy, Raman spectroscopy and XPS spectroscopy were used to analyze the reaction products. In hot (323 K) and acidic (pH 2) solutions under the presence of suspended iron(III) oxide hydroxide powder, DL-glyceraldehyde readily decomposes into low-molecular-weight compounds and transforms into sugar-like molecules via condensation reactions.

## 1. Introduction

A requisite for the emergence of life on the early Earth was the presence of water and organic compounds—primarily amino acids, nucleobases, sugars, and their respective precursors [1]. Sugars are considered one of the most essential molecules for all living organisms as they are vital in contemporary metabolism and the synthesis of other compounds, such as amino acids and nucleotides [2]. The previous functions of these biomolecules suggest that they were synthesized during the initial stages of the origin of life; therefore, to gain insight into the chemical processes that might have taken place on early Earth, investigating the mechanisms of abiotic sugar production is essential. The formose reaction, which involves the condensation of formaldehyde under basic conditions, is the most well-known of the reaction pathways in sugar synthesis and is dependent on the presence of inorganic catalysts, most commonly CaCO_3_ and Ca(OH)_2_ [3]. Alternative proposals for sugar synthesis include (A) anaerobic formose-like reactions with low-molecular-weight aldehydes in the presence of thiols [4] and ammonia catalysts [2,5], and (B) aldehyde condensation reactions catalyzed by mineral surfaces [3,6,7] Nevertheless, sugars are only by-products of these reactions, with the main organic compounds involving a wide variety of straight-chain and branched sugars and aldols. Since most of these reactions occur in environments that promote the breakdown of these organic compounds, studies must be carried out to propose alternate sugar synthesis reactions under plausible prebiotic conditions [1,8].

Researchers have proposed glyceraldehyde (C_3_H_6_O_3_) as an alternative to formaldehyde in the formose reaction for the synthesis of sugars, acting as a source of energy and monomers in aldehyde condensation reactions. In addition, glyceraldehyde can lead to sugar and sugar-like molecules through condensation reactions catalyzed by mineral surfaces [6] and possible nucleobases via reactions with ammonia under anaerobic conditions [9]. Alternatively, when glyceraldehyde is exposed to ionizing radiation, it acts as a source of molecules relevant to abiotic synthesis [10,11]. The decomposition products include glycolaldehyde, formaldehyde, malondialdehyde, and pyruvaldehyde. Further, glyceraldehyde can originate from both internal and external sources. Dihydroxyacetone, the ketone form of this aldehyde, may have reached early Earth through exogenous delivery from cometary and meteoritic debris [12,13]. Conversely, a by-product of formose-like processes, glyceraldehyde is produced internally and may have originated from endogenous sources in the Earth’s early environment [4].

All the previously discussed organic precursors could have been synthesized on Earth when the planet became habitable, as early as 10–20 million years after the Moon-forming impact [14]. Abiotic synthesis might have taken place in aqueous solutions in hydrothermal systems, in the atmosphere, or at the water–mineral interface of the Earth’s surface environments [14] during the Hadean (~4.54–4.0 Ga) and early Archean (~3.9 Ga) eons, when the planet’s atmosphere may have predominantly comprised CO_2_ [15,16]. Among these possibilities, hydrothermal systems have physicochemical traits that are believed to be crucial for the development of life [1]. Hydrothermal fluids are rich in ions, which can act as catalysts for chemical reactions and form metal complexes with organic molecules in solution [17]. Additionally, studies have revealed that hydrothermal systems provide concentration, redox, and temperature gradients that can be utilized for the abiotic synthetic processes of biomolecule precursors [1]. The thermal energy of these systems could also be used to start chemical reactions. Therefore, these geological environments are considered prime candidates for simulation experiments of abiotic synthesis under plausible early Earth geochemical conditions.

### The Kverkfjöll Volcano and the Hveradalur Geothermal Area, Central Iceland: Analog of an Ancient Hydrothermal System

**Figure 1:**
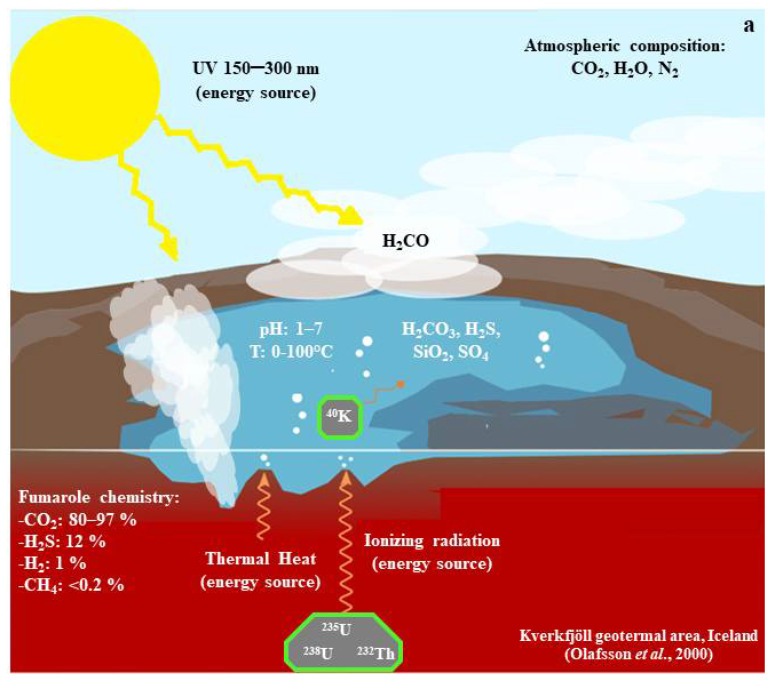
Schematic representation of an early Archean hydrothermal area. These surface hydrothermal environments could have been abundant during the Archean period (~3.9 Ga) in crustal zones with a high incidence of volcanic activity.

The Kverkfjöll volcano, located in Central Iceland, consists of a mountain massif of rocks of evolved basaltic composition, with an area of surface geothermal activity covering approximately 25 km^2^. This area displays a wide range of temperatures, pH values, and chemical compositions and has been extensively investigated [18]. Its western part, the Hveradalur geothermal area [19], is home to hydrothermal fluids of low pH (1–5) and low temperatures (<50 °C). This zone is of particular interest to the present work, acting as an analog of a low-temperature primitive early Archean (~3.9 Ga) hydrothermal area (Figure 1). Although representing a novel concept, using analog environments in prebiotic chemistry experiments shows great potential [13], as it allows researchers to reproduce the physicochemical conditions of geological environments that may have existed on ancient Earth. Analog environments also provide insight into the possible mineralogy of early Earth environments by allowing investigations into the distribution of mineral phases under the physicochemical conditions of interest.

Goethite (FeOOH), an iron(III) oxyhydroxide, is one of the mineral phases that potentially existed on primitive Earth and is of special relevance in studies involving the abiotic synthesis of organic molecules. It can act as a catalyst for aldehyde condensation reactions [6] and amino acid surface polymerization [20] and, therefore, possibly plays an important role in the abiotic synthesis of biomolecule precursors. Research has indicated that the sedimentary deposits of the early hydrothermal systems on Earth may have contained this mineral [21,22] Further, reference [19] reported the formation of goethite by hydrothermal modification of an iron meteorite under simulated hydrothermal conditions, thus demonstrating the mineral’s stability under conditions of low pH and high temperatures.

In the present work, we took quantitative measurements of remnant DL-glyceraldehyde in sample solutions after exposure to the simulated physicochemical conditions of an analog of a primitive Earth hydrothermal area, the Kverkfjöll geothermal field, using goethite as a catalyst for the mineral–water interface reactions. Through these measurements, we aimed to investigate the stability of glyceraldehyde and identify its decomposition products.

## 2. Materials and Methods

### 2.1. Reagents

All the reagents were of the highest commercial purity available, obtained from Sigma-Aldrich^®^, St. Louis, MO, USA, and included the following: DL-glyceraldehyde (>90%), acetaldehyde (>99.5%), pyruvaldehyde (40 wt. % in H_2_O), glyoxal (40 wt. % in H_2_O), glycolaldehyde (>90%), orcinol (>99%), D-glucose (99.9%), D-ribose (99.9%), and FeCl_3_ (95%). Methanol-free formaldehyde was prepared from paraformaldehyde according to the method described by [23]. Acetonitrile (HPLC), ethanol (HPLC), H_2_SO_4_ (95–97%), HCl (37 wt. % in H_2_O), HNO_3_ (70%), KOH (>85%), and 2,4-dinitrophenylhydrazine (DNPH) (97%) were obtained from Merck Co.^®^, Kenilworth, NJ, USA. Natural iron(III) hydroxide oxide samples were collected in Durango, Mexico, and were characterized by X-ray diffraction, Raman, and X-ray Photoemission spectroscopy (XPS). To eliminate organic contamination, the goethite samples were treated with a 3% HNO_3_ solution (acid wash), followed by two additional washes with Milli-Q^®^ (Merck Millipore, Burlington, MA, USA) water (deionized) and a 3% KOH solution. After the acidic and basic washes, the mineral samples were rinsed with Milli-Q^®^ water and dried in a desiccator at room temperature for 24 h.

### 2.2. Preparation of Samples

To simulate the Hveradalur geothermal site’s physicochemical conditions, a 1 × 10^−2^ mol/L, aqueous DL-glyceraldehyde solution was prepared using deionized water; the solution was acidified with 36 mM H_2_SO_4_ (pH 2). The samples were degasified with Ar to remove dissolved O_2_ in the solution. For the mineral–water interface reactions in the simulated environment, the system was mixed in 15-mL polyallomer centrifuge tubes with mineral (Goethite) powder (Beckman Coulter^®^, Brea, CA, USA). Goethite powder (180 mg), previously prepared from natural mineral samples by mechanical grinding in an agate mortar to an approximate grain size of 125 nm, was combined with 5 mL aliquots of the degasified acidic DL-glyceraldehyde standard solutions. The system was saturated with argon to create an anoxic atmosphere, and the polyallomer tubes were sealed to preserve the system’s atmospheric integrity. For the XPS measurements, small Goethite sheets (5 × 5 mm) were prepared. The sheets’ surfaces were polished with a SiC paste and cleaned with an additional acidic and basic wash. 

### 2.3. Sorption Experiments

The DL-glyceraldehyde/goethite samples were heated to 50 °C by placing the polyallomer tubes inside a water recirculation system. The experimental system was exposed to this thermal treatment at defined time intervals (120, 240, 360, and 480 min). After each time interval had elapsed, the mineral was separated from the aqueous phase using an Allegra XL-90 centrifuge (Beckman Coulter^®^, Brea, CA, USA) at 25,000 rpm at room temperature for 15 min. The goethite powder was desiccated and preserved for further analysis. The collected supernatants were filtered using 12-µm Acrodisc syringe filters (Whatman, Chicago, IL, USA) and stored at 0 °C for the subsequent HPLC measurements. The formation of new organic compounds in the sample supernatants was monitored by UV–Vis spectrophotometry, HPLC–UV, and HPLC-ESIMS; this sample analysis was repeated for each time interval used in the sorption experiments. For the sorption on goethite sheets, the same experimental procedure was repeated, combining the goethite sheet with 5 mL aliquots of DL-glyceraldehyde standard solutions, modifying the time intervals for the thermal treatment (300 and 600 min).

### 2.4. Carbonyl Compound Measurements

Using HPLC–UV, the aldehydes, as their 2,4-dinitrophenylhydrazine (DNPH) derivatives, were identified. The derivatization of the carbonyl compounds required reacting 5 mL aliquots of the sample supernatants with 5 mL aliquots of the DNPH reagent (0.4 mg DNPH dissolved in 2 mL H_2_SO_4_, 3 mL H_2_O, and 25 mL of ethanol) for 12 h. The carbonyl derivatives precipitated as yellow-orange crystals, which were filtered, dried, recrystallized, and redissolved in acetonitrile for subsequent analysis. The elution times of the carbonyl DNPH derivatives of formaldehyde, acetaldehyde, glyoxal, pyruvaldehyde, and DL-glyceraldehyde (as standard compounds) were measured.

### 2.5. Analysis of Samples

#### 2.5.1. HPLC–UV Analysis

For the HPLC–UV analysis of the carbonyl compounds, we used a Knauer^®^, Berlin, Germany) Azura P 4.1S HPLC pump equipped with a Knauer^®^ Azura UVD 2.1S UV detector. The derivatives of the carbonyl compounds were monitored by measuring their absorbance at 360 nm. The derivatives were separated in a Beckman^®^ Ultrasphere C-18 ODS column (250 × 4.6 mm) using isocratic elution at 1.0 mL/min with a mobile phase comprising 70% acetonitrile and 30% water. The elution times of the standard carbonyl–DNPH derivatives are presented in Table 1.

The elution times in the sample supernatants, which were unknown, were identified by comparing them with those of the standard aldehydes and carbohydrates. The quantitation of the remnant DL-glyceraldehyde in the experimental samples was determined by measuring the absorbance of DL-glyceraldehyde–DNPH at 360 nm as a function of time, employing the Beer–Lambert law to calculate the sample concentration. The calibration curve was constructed using the glyceraldehyde–DNPH standards, with a concentration interval of 1 × 10^−3^ to 1 × 10^−5^ mol/L.

#### 2.5.2. UV–Vis Spectroscopy

For the UV–Vis spectroscopic analysis, we used a Cary 100 spectrophotometer (Varian, Palo Alto, CA, USA). The supernatants of the DL-glyceraldehyde solutions after sorption were analyzed, monitoring their absorbance at 285 nm, which corresponds to DL-glyceraldehyde in solution. A concentrated (0.2 mol/L) solution was used to increase the sensitivity of the analyte to UV detection. 

#### 2.5.3. X-ray Diffraction Analysis

The purity of the natural goethite samples was measured by X-ray diffraction (XRD) using an Empyrean diffractometer (Malvern Panalytical^®^, Malvern, Worcs, UK) equipped with a PIXcel3D detector, with filtered Fe radiation of 60 kV at 2θ angles of 4° to 70°. The samples were prepared by grinding the goethite with an agate mortar to a particle size of less than <75 µm. The mineral powder was then pressed onto the diffractometer’s aluminum sample holder. The software HighScore (Malvern Panalytical^®^, Malvern, Worcs, UK), the Inorganic Crystal Structure Database (ICSD), and the International Center for Diffraction Data (ICDD) database were used for the mineral phase identification of the measured samples.

#### 2.5.4. Raman Spectroscopy Analysis

To study goethite and DL-glyceraldehyde, Raman spectroscopy was used to record their spectra. This analytical technique was also used to study the adsorption of organic compounds on the mineral surface. The experimental samples, in powder form, were pressed between two NaCl discs, and the Raman probe was used to collect the spectra of the samples. The Raman spectra were recorded using an Optosky ATR 3000 portable Raman spectrometer. The Raman probe containing a Class IIIB laser was positioned at an operating distance from the sample of 6 mm; the laser power was kept at a constant power value of 400 mW to avoid damage to the sample. The Raman frequencies were accurate to ±5 cm^−1^.

#### 2.5.5. XPS Analysis

The XPS spectra of the goethite mineral sample were recorded before glyceraldehyde adsorption and after adsorption for 300 and 600 min from the solution. The XPS analysis of the samples was carried out in an ultra-high vacuum chamber equipped with a hemispherical electron analyzer using an Al Kα X-ray source (1486.6 eV) with an aperture of 7 mm × 20 mm. The base pressure in the chamber was 5 × 10^−8^ mbar, and the experiments were performed at room temperature. The peak decomposition in different components was shaped, after background subtraction, as a convolution of Lorentzian and Gaussian curves. For deconvolution, we applied the criterion of using the lowest number of components for the fit. Binding energies were calibrated against the adventitious carbon component set to 285.0 eV for the goethite samples. The following core-level peaks were recorded under the same experimental conditions: C (1s), Fe (2 p_3/2_), and O (1s). We did not observe any beam radiation damage to the samples’ surfaces during the data acquisition.

#### 2.5.6. HPLC-ESIMS Analysis (Sugar-like Compound Measurements)

We define sugar-like compounds as molecules with five or six carbon atoms and multiple OH/CO substituents and isomers of carbohydrates. For the analysis of these molecules, HPLC–ESIMS was used. The analysis was carried out with a Waters^®^ 515 HPLC pump coupled with a Waters^®^ SQ-2 Single Quadrupole Mass Detector system, with electrospray ionization in negative (ESI-) mode. The cone voltage was 23 V, the capillary voltage was 1.46 kV, and the desolvation temperature was 350 °C. The sugar-like compounds were separated in a GL Sciences^®^, Torrance, CA, USA Inerstil NH_2_ 5 μm column (4.6 × 250 mm), specifically designed for the separation of carbohydrates, using isocratic elution at 0.8 mL/min with a mobile phase comprising 80% acetonitrile and 20% water.

## 3. Results

### 3.1. Goethite and DL-Glyceraldehyde Analysis

**Figure 2:**
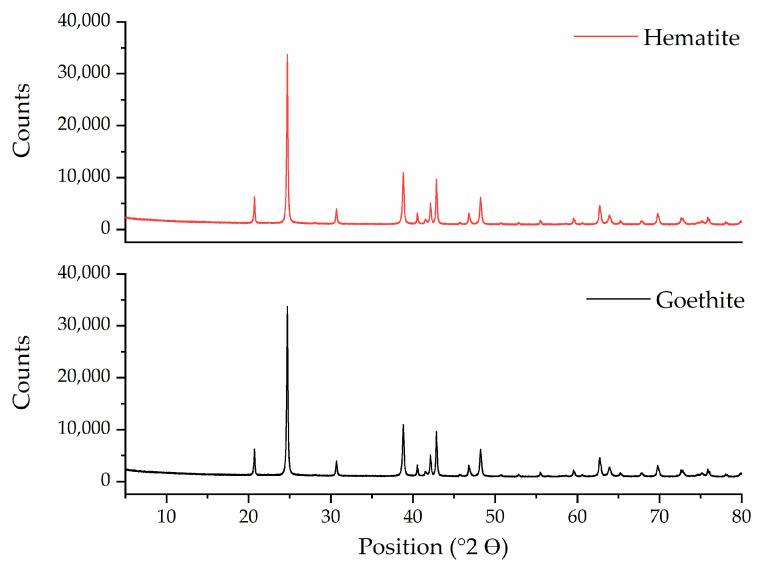
XRD pattern of collected natural goethite samples.

**Figure 3:**
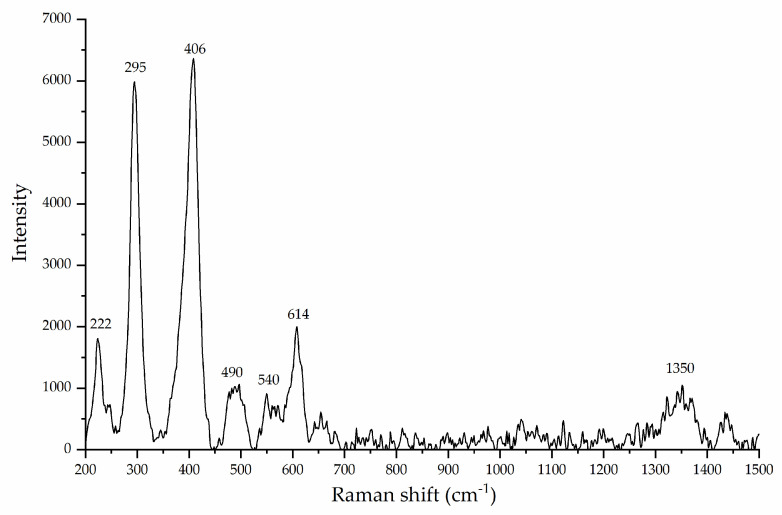
Raman spectrum of natural goethite mineral samples.

The XRD pattern (Figure 2) indicates, according to the ICSD and ICDD databases used for identification, the presence of two mineral phases in the collected samples: Goethite (91%) and hematite (9%), where goethite is the predominant mineral phase, as shown by the XRD pattern. The Raman spectrum (Figure 3) of the mineral sample powder shows vibrational bands associated with hematite and goethite mineral phases. The 222, 295, 406, 614, and 1350 cm^−1^ bands belong to hematite. Regarding goethite, the reported vibrational bands were 243, 299, 385, 479, 550, 685, and 993 cm^−1^ [25]. In the present spectrum, the 299, 385, and 479 cm^−1^ vibrational bands overlap with the hematite bands, whereas the peaks at 685 and 993 cm^−1^ were not present. The Raman spectrum confirms that the mineral sample is a mixture of goethite and hematite. Hematite is present as a minor constituent of the mineral phase.

**Figure 4:**
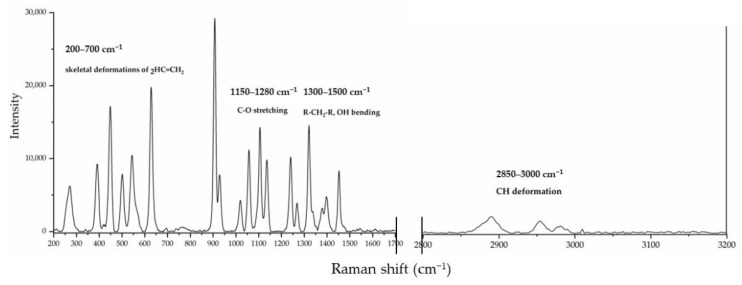
Raman spectrum of DL-glyceraldehyde powder.

For DL-glyceraldehyde, the Raman spectrum shows that commercial (Sigma-Aldrich^®^) DL-glyceraldehyde isomers are prevalent when the molecule is in the crystalline state. Figure 4 shows the vibrational bands and their associated functional groups as reported in previous works [26]. The vibrational band associated with the carbonyl bond (1700 cm^−1^) is not detected in the experimental samples. However, multiple peaks in the region of 200 to 700 cm^−1^ were found on the experimental samples. These vibration bands are not reported in the literature The last optical activity in the 200–700 cm^−1^ region is associated with the multiple deformations of an alkene (C=C) group [27]. This functional group is present on the isomer of DL-glyceraldehyde present in the sample, an enol.

### 3.2. Iron(III) Oxide Hydroxide in a Simulated Archean Hydrothermal Area

**Figure 5:**
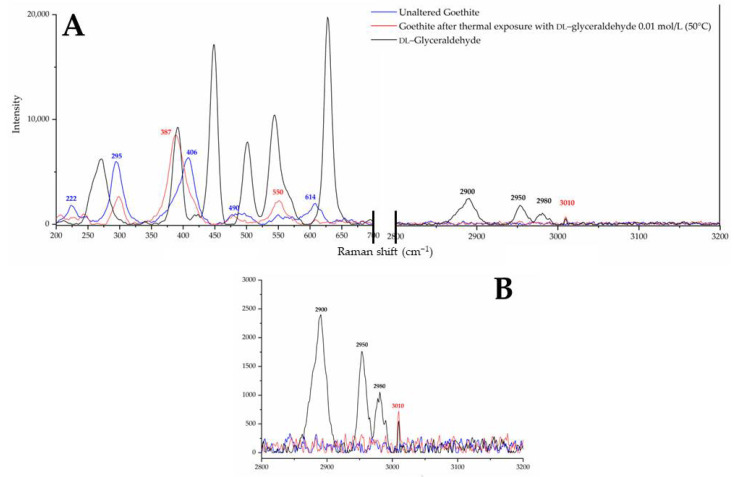
(**A**) Raman spectra of unaltered goethite (blue line) compared to altered goethite (red line) and DL-glyceraldehyde. Two vibrational bands of altered goethite overlap with bands associated with DL-glyceraldehyde in the 200–700 cm^−1^ region A sole band overlaps in the 2500–3500 cm^−1^ region. (**B**) 2800–3200 cm^−1^ region of the Raman spectra at a reduced-intensity scale.

Iron(III) oxide hydroxide in its crystalline state was analyzed by Raman spectroscopy before and after sorption with aqueous DL-glyceraldehyde (Figure 5). Analysis of the region between 200 and 700 cm^−1^ reveals the overlapping of the 406 and 550 cm^−1^ vibrational bands of the goethite/DL-glyceraldehyde system with those corresponding to the skeletal deformations of alkenes (C=C) (Figure 5A). Using the Raman spectrum of unaltered crystalline goethite as a reference, the increase in the intensity of the 550 cm^−1^ band is notable. Additionally, the width of the 387 cm^−1^ band increase suggests that it is composed of at least two components, one of these being the 406 cm^−1^ vibrational band of DL-glyceraldehyde. An additional overlapping band is present in the region of 2500–3500 cm^−1^ (Figure 5B), associated with CH vibrations. As mentioned earlier, these vibrational bands in this region correspond to the enol form of glyceraldehyde.

**Figure 6:**
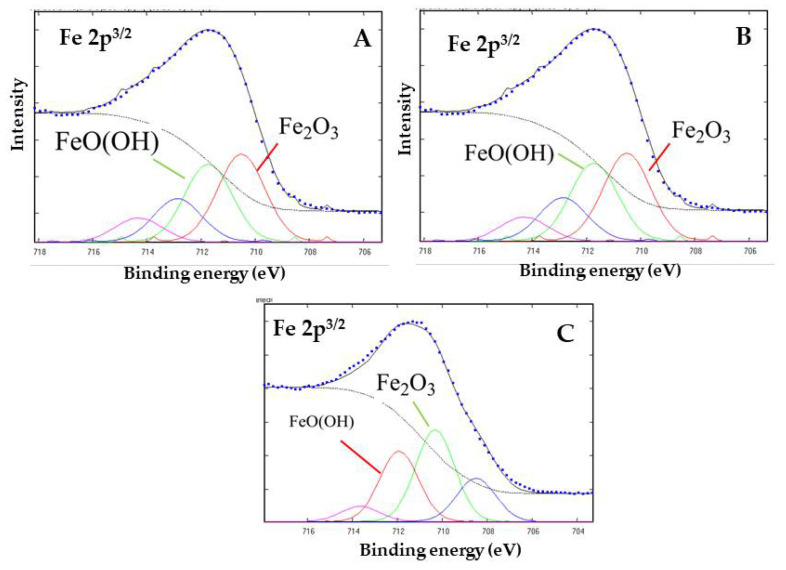
XPS core level peak of Fe 2p_3/2_ for goethite after absorption of 0.01 mol/L DL-glyceraldehyde solution, heated at 50 °C for 300 (**A**) and 600 (**B**) minutes. The spectrum of goethite samples before absorption (**C**) is shown as reference. The spectra consist of experimental (…) and fitted components (color lines).

To obtain additional information and confirmation regarding the adsorption and chemical forms of the organic constituents on the surface of goethite, the mineral was characterized by XPS spectroscopy before and after the sorption of aqueous DL-glyceraldehyde at defined time intervals (300 and 600 min). The Fe 2p_3/2_, C 1s, and O 1s peaks have been deconvoluted into multiple component curves, representing the best fit obtained for these regions. Figure 6 shows the deconvoluted core level spectra of Fe 2p_3/2_. The binding energies of the two main components of the Fe 2p_3/2_ peak are located at 711.9 and 710.3 eV, corresponding to FeO(OH) (goethite) and Fe_2_O_3_ (hematite), respectively. (Figure 6A–C). The remaining Fe component curves are associated with different iron species (such as metallic Fe and multiple oxides species).

**Figure 7:**
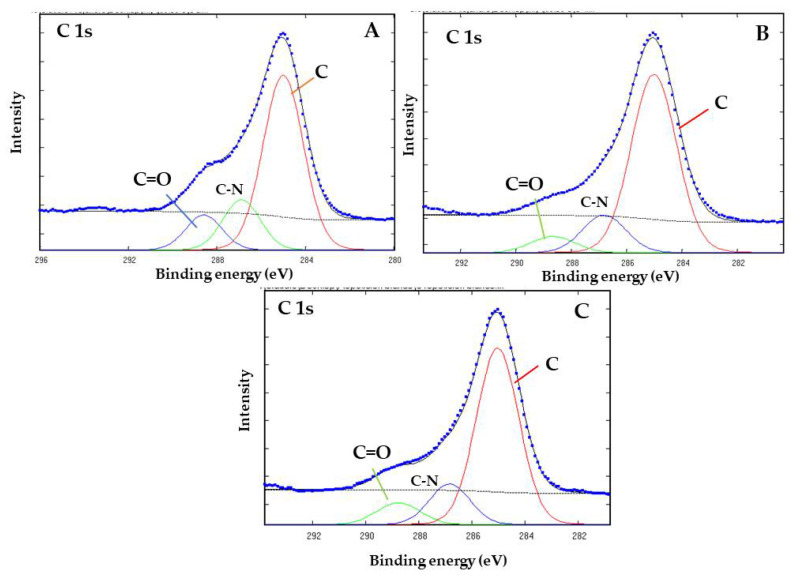
XPS core level peak of C 1s for goethite after adsorption of 0.01 mol/L DL-glyceraldehyde solution, heated at 50 °C for 300 (**A**) and 600 (**B**) minutes. The spectrum of goethite samples before absorption (**C**) is shown as reference. The spectra consist of experimental (…) and fitted components (color lines).

As for the deconvolution of the core level spectra of C 1s, Figure 7 shows the binding energies of the three components of the C 1s peak—285.0, 286.6, and 288.5 eV, which correspond to C-H, C-C, and adventitious carbon, the second one to C-N, and the third one to carboxyl (C=O) compounds, respectively [28]. The 285.0 and 286.6 eV binding energies are associated with compounds formed by hydrocarbon and nitrogen impurities present in the original mineral sample (Figure 7C). The carboxyl compound component was also present in the unaltered mineral sample. After 300 min of thermal exposure to the organic component, the intensity of the component increases (Figure 7A); then, with increased thermal exposure time (6000 min), the intensity of the component decreases (Figure 7B).

**Figure 8:**
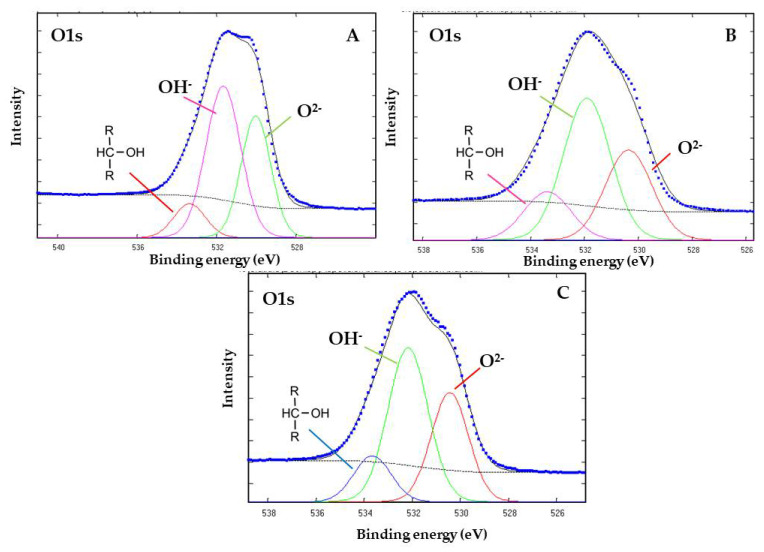
XPS core level peak of O 1s for goethite after adsorption of 0.01 mol/L DL-glyceraldehyde solution, heated at 50 °C for 300 (**A**) and 600 (**B**) minutes. The spectrum of goethite samples before absorption (**C**) is shown as reference. The spectra consist of experimental (…) and fitted components (color lines).

Figure 8A shows the deconvoluted core level spectra of O 1s. The binding energies of the three components of the O 1s peak were 530.4, 531.8, and 533.3 eV. The 530.4 eV component corresponds to the OH^−^ and oxides species. The component at 531.8 eV corresponds to the adventitious oxygen and O^2−^ surface atoms. This is coherent with the goethite structure (FeO(OH)). The peak at 533.3 eV corresponds to an organic compound with the functional group RC-OH [28]. The 533.3 eV binding energy intensity increases slightly when the thermal exposure time of the goethite/aqueous DL-glyceraldehyde mixture was increased to 600 min (Figure 8B). The 533.3 eV binding energy is present in the unaltered goethite mineral samples (Figure 8C).

**Figure 9:**
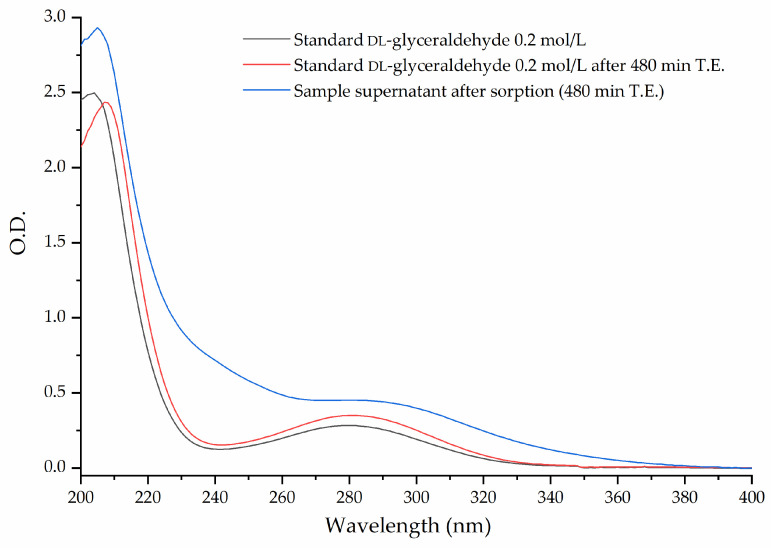
Comparison of the UV spectra of stock DL-glyceraldehyde solution (black line), stock DL-glyceraldehyde solution heated at 50 °C up to 480 min (red line), and underivatized supernatant analyzed after sorption (blue line).

To determine whether the mineral phase or the temperature is responsible for the decomposition of DL-glyceraldehyde, the UV spectrum of the underivatized supernatant was analyzed by UV-Vis spectroscopy to monitor the absorption of the 285 nm peak after sorption at the maximum time of thermal exposure (Figure 9). The spectrum was compared to a control sample of the DL-glyceraldehyde solution heated at a constant temperature of 50 °C for 480 min. No notable change in the absorption spectra was observed between the DL-glyceraldehyde standard and the heated sample. However, an increase in overall absorption and a shift to higher wave numbers of the 285 nm band was discerned when the sample was heated in the presence of goethite.

### 3.3. Stability of DL-Glyceraldehyde in a Simulated Archean Hydrothermal Area

**Figure 10:**
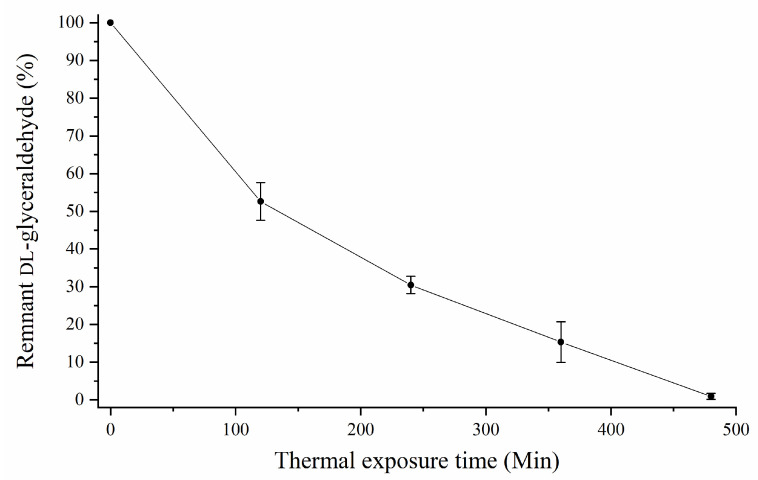
Percentage of remnant DL-glyceraldehyde in aqueous solution as a function of thermal exposure time (at 50 °C).

**Figure 11:**
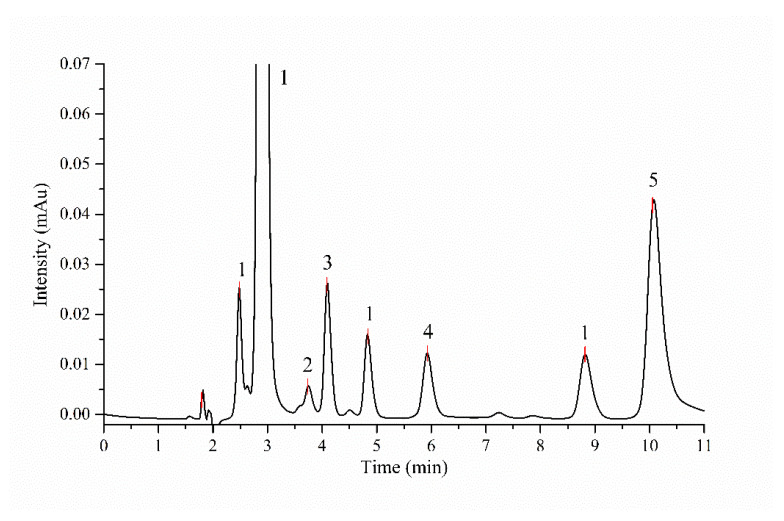
HPLC chromatogram of sample supernatant derivatized with DNPH after 120 min of thermal exposure: (1) Glyceraldehyde-DNPH. (2) Formaldehyde-DNPH. (3) Acetaldehyde-DNPH. (4) Unknown DNPH. (5) Pyurvaldehyde-diDNPH. Peaks marked with 1 are associated with Glyceraldehyde-DNPH standard (Figure 12).

**Figure 12:**
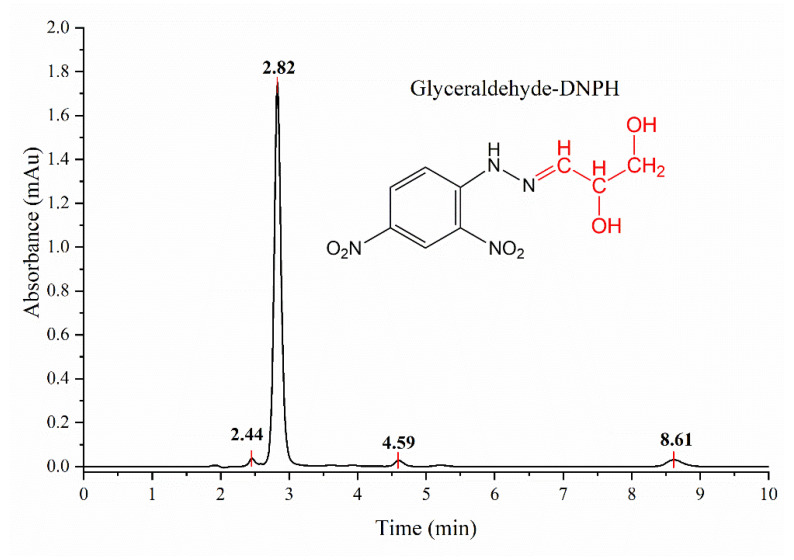
HPLC chromatogram of Glyceraldehyde-DNPH standard.

**Figure 13:**
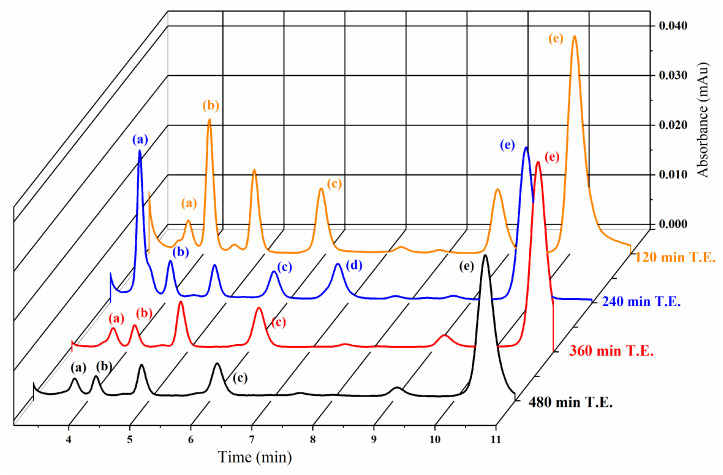
HPLC chromatogram of sample supernatants derivatized with DNPH at different times of thermal exposure (T.E). (a) Formaldehyde-DNPH. (b) Acetaldehyde-DNPH. (c) Unknown carbonyl-DNPH. (d) Glyoxal-diDNPH. (e) Pyurvaldehyde-diDNPH. Retention times of the samples exposed to 120 and 240 min of thermal exposure are slightly displaced when compared with the retention times of the samples exposed to 360 and 480 min of thermal exposure.

HPLC analysis shows that DL-glyceraldehyde is labile under the simulated hydrothermal conditions (Figure 10). The total percentage of remnant DL-glyceraldehyde in the solution decreases at higher thermal exposure times. The decomposition products are formaldehyde, acetaldehyde, glyoxal, and pyruvaldehyde. An unknown decomposition product, which could not be identified with the available carbonyl-DNPH standards, with an elution time of 5.9 min was also detected (Figure 11). The chromatogram of the DL-glyceraldehyde standard, derivatized with DNPH, shows the presence of multiple compounds (Figure 12) associated with the presence in solution of multiple isomers formed by keto-enol tautomerism of the sample. As for the unknown decomposition product, the HPLC mobility of the DNPH derivatives suggests that this compound could be glyceric acid, a previously identified decomposition product of DL-glyceraldehyde in an aqueous solution [11]. The decomposition products were stable for up to 480 min of thermal exposure (Figure 13).

### 3.4. Mass Spectrometric Detection of Putative C5 and C6 Sugar-like Compounds

**Figure 14:**
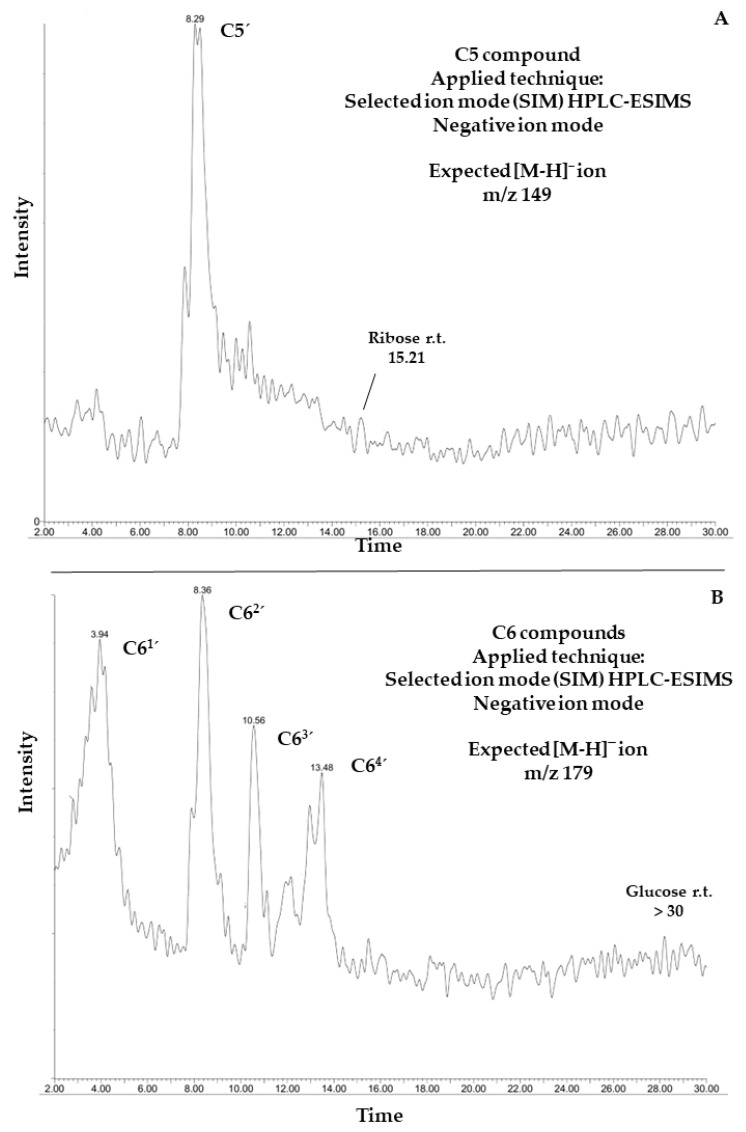
HPLC-ESIMS for C5 and C6 compounds detected in sample supernatants of DL-glyceraldehyde sorption in a simulated Archean hydrothermal area. (**A**): HPLC-ESIMS of the C5′, an organic compound with a molecular weight of 150 g/mol. (**B**): HPLC-ESIMS of four organic compounds (C6^1^′, C6^2^′, C6^3^′, and C6^4^′), each one of them with a molecular weight of 180 g/mol. Retention times of ribose and glucose are shown as reference.

HPLC-ESIMS (Figure 14) was used for the detection of C5 and C6 (sugar-like) molecules in the sample supernatants, analyzed after the sorption with goethite. The applied technique was selected ion mode (SIM) HPLC-ESIMS monitoring. In negative ion mode, expected [M–H]^−^ ions were registered; *m*/*z* 149 for pentoses and *m*/*z* 179 for hexoses. Five compounds (C5′and C6^1^′, C6^2^′, C6^3^′, and C6^4^′) were detected with a molecular weight of 150 (C5′) (Figure 14A) and 180 g/mol (C6^1–4^′) (Figure 14B). These organic compounds were detected in all the sorption samples at all thermal exposure time intervals. Figure 14 shows the chromatogram of the supernatant exposed to 360 min of thermal exposure The retention times of these organic compounds were compared to the retention times of two carbohydrate standards (ribose and glucose). Although nominal *m*/*z* ratios of detected compounds coincide with those of ribose and glucose, the corresponding retention times are different. This suggests that the detected organic compounds are isomers of ribose and glucose, with molecular weights of 150 and 180 g/mol, respectively.

## 4. Discussion

**Figure 15:**
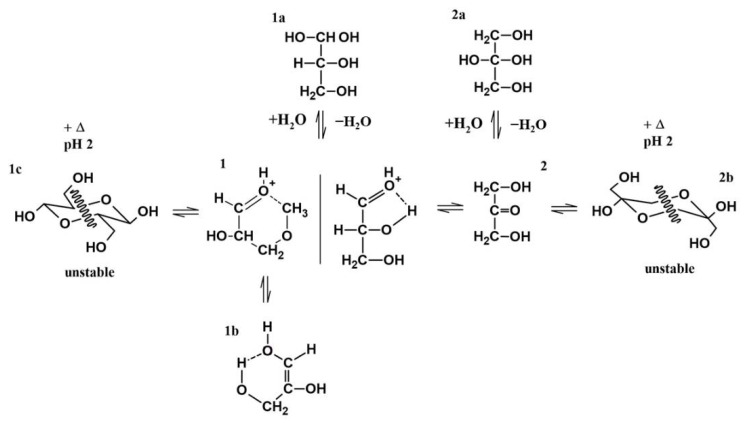
Proposed molecular interconversions of DL-glyceraldehyde in acid aqueous solution. (**1**) DL-glyceraldehyde (C-2 OH H bond), DL-glyceraldehyde (C-3 OH H bond). (**1a**) Hydrated glyceraldehyde. (**1b**) DL-glyceraldehyde enediol. (**1c**) Glyceraldehyde cyclic hemiacetal. (**2**) Dihydroxyacetone. (**2a**) Hydrated dihydroxyacetone. (**2b**) Dihydroxyacetone cyclic hemiketal.

**Figure 16:**
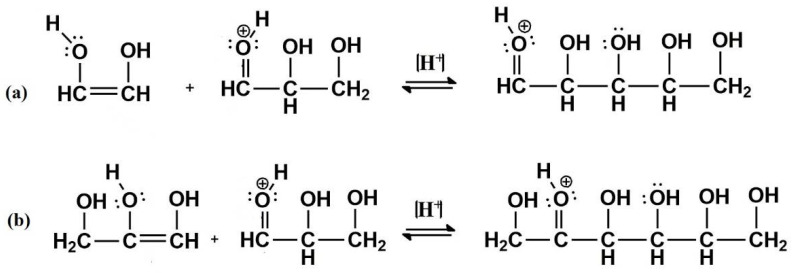
Proposed reaction mechanism for C5 and C6 compounds. Equation (**a**): Pentose isomer formation (aldol condensation of glyoxal and DL-glyceraldehyde). Equation (**b**): Hexose isomer formation (aldol condensation of DL-glyceraldehyde isomers).

Investigations into the stability of DL-glyceraldehyde in aqueous solutions have seldom been conducted, and those that have focused on the role of this organic compound as a source of aldehyde monomers in autocatalytic reactions in sugar synthesis [5,6,9] and its stability under high-energy radiation fields in simulated prebiotic environments [10,11]. The novelty of the current work lies in the insight provided into the possible role of DL-glyceraldehyde as a source of aldehydes and C5/C6 compounds in simulated hydrothermal surface areas, where acidic conditions and variable temperatures are dominant. DL-glyceraldehyde in solution undergoes interconversion between different chemical species that exist in a complex equilibrium [24]. Under the experimental conditions, the dimeric cyclic hemiacetal forms of DL-glyceraldehyde are unstable; consequently, the keto and enol species are dominant (Figure 15). Intramolecular hydrogen bonds between the carbonyl carbon and the C-2 and C-3 hydroxyl hydrogens (1) stabilize the ketone form, shifting the equilibrium toward these species and ensuring that the enediol (1b) and hydrated forms (1a) are not dominant in solution. Therefore, it is highly probable that these molecules are not the dominant form of glyceraldehyde present in the solution. This chemical equilibrium is ubiquitous, regardless of whether the sample is in crystalline form or solution [24]. The Raman spectrum shows that crystalline DL-glyceraldehyde exists as the enol form (Figure 16, 1b), which contains the functional group C=C. The UV absorption peak of 285 nm (Figure 9) indicates the presence of this isomer in the solution. [29]. Derivatization with DNPH of DL-glyceraldehyde in solution shows that the carbonyl isomer is formed when the crystalline form is dissolved in water (Figure 12). Due to the complex keto enol tautomerism of the sample in solution, additional carbonyl compounds are present. However, the concentration of the impurities is low enough when compared to DL-glyceraldehyde-DNPH.

The decomposition products of DL-glyceraldehyde, when under acidic thermal conditions, are formaldehyde (CH_2_O), acetaldehyde (C_2_H_4_O), glyoxal (C_2_H_2_O_2_), and pyruvaldehyde (C_3_H_4_O_2_) (Figure 11). Additionally, one C5 and four C6 compounds, isomers of pentose and hexose with a molecular weight of 150 and 180 g/mol, respectively, were detected in the experimental samples (Figure 14). The formation of these isomers can be explained by the acid-catalyzed aldol condensation of the aldehydes with their respective enol forms. The protonated DL-glyceraldehyde reacts via the addition of 1,2-enediol (glyoxal isomer), leading to an aldol condensation product, a pentose isomer (Figure 16a). The addition of the keto and aldol forms of DL-glyceraldehyde can yield hexose isomers as condensation products (Figure 16b).

The instability of this molecule in the experimental conditions is due to the displacement of the hemiacetal equilibrium. In the presence of heat and aqueous acid, the formation of hemiacetals, which act as protective chemical groups of carbonyl compounds through the reduction of their reactivity, is hindered. This process increases the reactivity of the linear carbonyl compounds in solution, which, therefore, promotes the synthesis of different organic compounds. Nevertheless, the aldol condensation reaction is not selective to specific C5 and C6 compounds, and multiple products could form via crossed aldol reactions (i.e., condensation between two different aldehydes/ketones [30]). This is demonstrated by the detection of the C5′ and C6^1–4^′ compounds (Figure 14) which are isomers of a pentose and a hexose. The lack of selectivity towards specific carbohydrates will yield a mixture of sugar-like compounds. The initial concentration of the stock solution hinders the formation of these compounds. C5′ and C6^1–4^′ were detected only when the concentration of the stock DL-glyceraldehyde solution was increased to 0.2 mol/L, up from 0.01 mol/L. A high concentration of the initial aldehyde is required to obtain high yields of the C5′ and C6^1–4^′ compounds. Therefore, the total yield of this compound must be low. In a hot, acidic environment, most of the DL-glyceraldehyde readily decomposes into low-molecular-weight aldehydes (<90 g/mol). Further analysis with higher-resolution techniques is required to reveal the structural features of the detected compounds. 

Previous studies have investigated the role of goethite in carbohydrate synthesis. For example, reference [6] proposed the formation of C6 compounds via the catalytic action of goethite, which occurs through the aldol condensation of DL-glyceraldehyde with a mineral-stabilized glyceraldehyde enediolate on the surface of the iron(III) oxide hydroxide. Exhaustive analysis of the peak frequencies in goethite’s Raman spectrum after exposure to solutions of DL-glyceraldehyde 0.01 mol/L (Figure 5) shows that two of the vibrational peaks (406 and 550 cm^−1^) of goethite overlap with the vibrational peaks of DL-glyceraldehyde, which are associated with skeletal deformations of the C=C bond of the triose enol form. In particular, the 550 cm^−1^ band of goethite increases in intensity, while the 406 cm^−1^ band widens. These changes in the Raman spectrum suggest the adsorption of a DL-glyceraldehyde isomer (enol) on the surface of goethite. Regarding the XPS data, two binding energies are of special interest: 288.5 eV of core level C (1s) and 533.3 eV of core level O (1s). The increase in the intensity of the 288.5 eV component (associated with a carboxyl group) when the thermal exposure time is increased to 300 min can be explained by the adsorption of a carbonyl compound on the surface of goethite. The decrease exhibited when the thermal exposure time is further increased could be attributed to the decomposition of the same molecule. Due to the keto-enol equilibrium and decomposition of DL-glyceraldehyde (Figure 15), the adsorbed molecule could be either DL-glyceraldehyde, dihydroxyacetone, or one of the aldehydes formed by its decomposition (Figure 11). Regarding the 533.3 eV component, the increase in the intensity, proportional to the increase in thermal exposure time, can be explained by the gradual adsorption of RCOH-rich compounds on the mineral surface or by the decomposition compounds, which will continue to contribute to the increase in the same oxygen component (C-O, C-O-H). However, the exact chemical formulas of these compounds remain unknown. Considering the molecules formed in the solution due to the keto-enol tautomerism and decomposition of DL-glyceraldehyde, the adsorbed molecules could be either C5/C6 compounds, an enol isomer, an aldehyde, or hydrated glyceraldehyde/dihydroxyacetone.

According to the literature [6], the adsorption of DL-glyceraldehyde on the surface of goethite occurs due to the formation of an Fe^3+^-O_2_R bond between two OH groups of the glyceraldehyde enol isomer. However, neither the Raman nor XPS spectrum shows evidence of vibrational peaks associated with the formation of an organometallic bond. Therefore, the exact mechanism by which the organic compounds are adsorbed on the surface of the goethite samples remains elusive.

The existence of Fe^3+^ over the reduced Fe^2+^ form in an acidic aqueous environment can be explained by aqueous corrosion mechanisms, which can occur in both oxygenic and anoxic environments [31]. In the presence of oxygen, goethite (and Fe^3+^ oxyhydroxides) can form via the corrosion of Fe/Ni deposits or iron meteorites. Fe^0^ can be oxidized by two oxidizing agents, O^2^ and H^+^, degrading the initial material into α- and β-FeO(OH) [32]. In acidic deaerated solutions, protons act exclusively as the oxidizing agent. Previous works provided evidence of the formation of hematite and goethite in an anaerobic acid environment [19,33], which confirms the ability of hot, low-pH environments to oxidize Fe^0^ to Fe^3+^. Goethite (and hematite) are considered the end members of many iron transformation routes due to their thermodynamic stability. Therefore, conversion to additional iron mineral phases is not expected [32].

### Implications for Prebiotic Chemistry

Under the simulated hydrothermal conditions, formaldehyde, acetaldehyde, glyoxal, and pyruvaldehyde form via the thermal decomposition of DL-glyceraldehyde. Formaldehyde is considered the cornerstone organic molecule for carbohydrate synthesis via the formose reaction [3]. Strecker synthesis with acetaldehyde and ammonia can be used as a source for alanine [33] while glyoxal and pyruvaldehyde are usually categorized as intermediates in formose-like reaction pathways [4]. The results of the current work show that DL-glyceraldehyde could have been a prebiotic source of monomers in primitive hydrothermal environments, molecules that, under variable pH conditions, could act as energy sources for amino acid and carbohydrate synthesis [34]. Nevertheless, the aldol condensation of DL-glyceraldehyde provides an additional source of C5 and C6 compounds in hydrothermal environments. The possible pathway that can lead to the synthesis of these organic compounds is the acid-catalyzed aldol condensation between DL-glyceraldehyde and enol molecules. However, the yield of these acid-catalyzed aldol condensation reactions is low.

As mentioned earlier, researchers generally believe that the prebiotic synthesis of carbohydrates and carbohydrate precursors involved the condensation of low-molecular-weight aldehydes under basic conditions (e.g., pH > 11) in the presence of inorganic catalysts [1,13]. The synthesis of C5 and C6 compounds under acidic conditions seems notably more plausible than the reaction pathways commonly proposed, as surface hydrothermal systems were common during the early Archean [15,35]. Regarding goethite, prebiotic sources of this mineral could have arisen from the photooxidation of dissolved Fe(II), [6] and aqueous alterations of iron-rich rocks and minerals [19,22]. Therefore, it is highly likely that this mineral was common in the sediments of ancient hydrothermal regions [20,21].

## 5. Conclusions

Prebiotic carbohydrate synthesis is fundamental because of its role in metabolism and the abiotic formation of nucleotides and nucleic acid components. In this work, we investigated the stability of DL-glyceraldehyde in a simulated Archean hydrothermal environment and reached the following conclusions:

1. DL-glyceraldehyde readily decomposes in the presence of goethite under simulated hydrothermal conditions, and the decomposition products can react with remnant glyceraldehyde in an acid-catalyzed aldol condensation reaction to generate C5 and C6 compounds.
2. The thermal decomposition products of glyceraldehyde (formaldehyde, acetaldehyde, glyoxal, and pyruvaldehyde) are molecules of prebiotic significance; they are precursors of carbohydrates in formose-like reactions and other organic compounds.
3. Enol, aldehydes, and C5/C6 compounds, products of the decomposition of DL-glyceraldehyde in a simulated Archean hydrothermal area, successfully bind to the goethite surface.
4. C5 and C6 compounds could form through aldol condensation in an aqueous solution. These compounds are stable under acidic and thermal conditions for brief periods of time. The presence of the sugar-like compounds, C5′ and C6^1–4^′, implies that a rather complex mechanism of sugar-like compound formation occurs in the experimental system. These reaction mechanisms are not selective towards specific pentose and hexoses, such as ribose or glucose.

## Figures and Tables

**Table 1:** Elution times of standard carbonyl-DNPH derivatives.

Beckman^®^ Ultrasphere C-18 ODS Column.	
Compound	Elution time (min)
Glyceraldehyde-DNPH	2.44, 2.82, 4.59, 8.61 *
Glycolaldehyde-DNPH	3.0
Formaldehyde-DNPH	3.62
Acetaldehyde-DNPH	4.13
Glyoxal-diDNPH	7.25 ± 0.7
Pyruvaldehyde-diDNPH	10.64

* Multiple retention times of the DL-glyceraldehyde standard are possibly associated with multiple compounds formed by the keto-enol tautomerism of the molecule in water [24].

## Data Availability

All experimental data in this study will be made available upon reasonable request from readers.
